# Supreme Court Decision

**Published:** 1881-01

**Authors:** James H. McKenney

**Affiliations:** Clerk Supreme Court U. S.


### SUPREME COURT OF THE UNITED STATES.

No. 67. October Term, 1880.

The Goodyear Dental Vulcanite Company, Appellant, *vs.* Charles G. Davis.

Appeal from the Circuit Court of the United States for the District of Massachusetts.

Mr. Justice Strong delivered the opinion of the Court.

The invention described in the Cummings re-issue patent is claimed in the words following: "The plate of hard rubber or vulcanite, or its equivalent, for holding artificial teeth, or teeth and gums, substantially as described." The claim cannot be understood without reference to the details given in the specification. In that it is said to consist "in forming the plate to which the teeth, or teeth and gums, are attached, of hard rubber, or 'vulcanite,' so called — an elastic material possessing and retaining in use sufficient rigidity for the purpose of mastication, and, at the same time, being pliable enough to yield a little to the motions of the mouth." The mode of "forming" the plate is then minutely described. The earlier steps of the process need not be particularly noticed. They relate to the formation of a plaster mould, fitted to the corresponding part of the mouth, with the artificial teeth adhering in the mould in exactly the relative position they are to occupy in the hard-rubber plate. The specification then proceeds as follows: "The teeth are provided with pins projecting therefrom in such a manner that the rubber which is to constitute the plate will close around them, and by means of them hold or secure the teeth permanently in position. The plaster mould, with the teeth adhering therein, as just described, is now filled with soft rubber, a little at a time, pressed in with the finger, or in any other convenient way; and care is to be taken that the rubber is made to completely fit into the cavities and around the protuberances, including the pins, and is filled in to the thickness or depth desired to form the plate." "I then" (says the patentee) "lock the rubber plate in position by shutting the other half of the plaster mould over it, to insure its retaining its exact form while warming, and then heat or bake it in an oven, or in any other suitable way. The soft rubber or gum so inserted into the mould, is to be compounded with sulphur, rubber, &c., in the manner prescribed in the patent of Nelson Goodyear, dated May 6, A. D. 1851, for making hard rubber, and is to be subjected to sufficient heat to vulcanize or harden it, substantially as directed in that patent. It is also to be colored in imitation of the natural gums, by mixing it with vermilion, or other suitable coloring matter, while in the soft state. After the plate has been heated sufficiently to harden it. or convert it into hard rubber or vulcanite, so called, the mould is removed, and the plate is polished ready for use."

Such is the description both of the material of which the plate is formed, and of the method or process by which it is made.

We had occasion in Smith *vs.* Goodyear Dental Vulcanite Company *et al.*, 93 U. S. Rep., 486, to construe this patent, and determine what the invention claimed and patented really was. We held it to be "a set of artificial teeth, as a new article of manufacture, consisting of a plate of hard rubber with teeth, or teeth and gums secured thereto in the manner described in the specification, by embedding the teeth and pins in a vulcanizable compound, so that it shall surround them while it is in a soft state, before it is vulcanized, and so that when it has been vulcanized the teeth are firmly and inseparably secured in the vulcanite and a tight joint is effected between them, the whole constituting but one piece." We said "the invention is a product or manufacture made in a defined manner. It is not a product alone, separated from the process by which it is created." The process detailed in the description antecedent to the claim, and referred to thereby, is as much a part of the invention as are the materials of which the plate or product is composed. Both are necessary elements of it. Hence, to constitute an infringement of the patent, both the material of which the dental plate is made, or its equivalent, and the process of constructing the plate or a process equivalent thereto, must be employed. It is, therefore, essential to a correct determination of this case, to consider what was the material made by the patentee an element of his invention, and what can be considered an equivalent therefor.

It is impossible to read the specification of the original patent, or that of the re-issue, upon which this suit is founded, without the conviction that the patentee had in mind primarily a single substance for his material, and that one of a peculiar character, itself a compound discovered and patented not long before. Thus, in the original, which was loosely drawn, the invention was said to consist "in forming the plate and gums, to which the teeth are attached, of rubber, or some other elastic material, so indurated as to be rigid enough for the purpose of mastication, and pliable enough to yield a little to the motions of the mouth, and in one piece, the teeth being embedded in the elastic material while the said material is in a soft condition, and then baked, with the gums and plate, so that the teeth, gums and plate will all be connected, forming, as it were, one piece." And again, "the plate and gums are formed of one piece, and of rubber, and the compounds commonly employed therewith, or of gutta-percha, or, in fact, of any elastic substance which can be reduced to a soft condition, *and then vulcanized*, or hardened sufficiently to answer the purpose. The rubber or other material used is first moulded to fit the shape of the mouth, and the gums formed, and while soft and pliable the teeth are embedded in the gums. . . . The teeth, gums, and plate, being thus connected, are then baked until the elastic material becomes sufficiently vulcanized, when the process is completed." The claim also expressed the same thought. It was "forming the plate and gums in which the teeth are inserted, in one piece of hard rubber or vulcanite, *i. e.*, an elastic material which can be hardened sufficiently for mastication, and retain a portion of its elasticity, so as to yield a little to the motion of the mouth, as herein set forth, and for the purposes specified." Though the specification spoke of rubber, or rubber and its compounds commonly employed therewith, or of gutta-percha, or of any elastic substance that can be reduced to a soft condition and then vulcanized, or hardened sufficiently to answer the purpose, it is evident only such substances were intended as are either soft in their natural condition, and capable of vulcanization, or if hard naturally and reduced to a soft state, were then capable of being vlcanized or hardened by baking. The description implies an exclusion of all substances which have not such capabilities. If not, then gold plates would have been within the patent, if the material only be considered, for gold is elastic, when in thin plates. It is capable of being reduced to a soft condition, and it hardens by cooling, but it does not harden by heat or baking, nor is it capable of vulcanization.

If, now we turn to the specification and claim of the re-issued patent, (which, of course, cannot be more comprehensive than the original,) it will be found that the material is still more distinctly and exclusively pointed out. The material is, so far as possible, restricted to a substance either vulcanized or capable of vulcanization. There the invention is said to consist "in forming the plate to which the teeth, or teeth and gums, are attached, of hard rubber or vulcanite, so called, an elastic material," of certain capabilities mentioned. Not an intimation is given that any other substance than hard rubber, or its synonym, vulcanite, would meet the requirements of the invention. Throughout the specification the patentee speaks again and again of his invention as a hard-rubber plate, and he describes minutely the process or mode of making it. After having mentioned the arrangement of the teeth in a mould, he says: "The plaster mould, with the teeth adhering therein, is now filled with soft rubber, a little at a time, pressed in with the finger, or in any other convenient way," &c., &c., &c. "I then lock the rubber plate in position by shutting the other half of the plaster mould over it, to insure its retaining its exact form while warming, and then heat or bake it in an oven, or in any other suitable way. The soft rubber, or gum, so inserted in the mould, is to be compounded with sulphur, rubber, &c., in the manner prescribed in the patent of Nelson Goodyear, dated May 6, A. D. 1851, for making hard rubber, and is to be subjected to sufficient heat to vulcanize or harden it, substantially as directed in that patent." Thus it appears that, whatever the material used in the construction, a dental plate, according to the patent, was required to undergo a process of vulcanization in the manufacture, and to be made what it is, whether denominated rubber, hard rubber, vulcanite, or other elastic material, by that process. Every substance not capable of vulcanization by that process was, therefore, necessarily excluded from the reach of the patentee's invention. Nothing could more plainly show that a dental plate made of any other material than a compound, vulcanized according to Nelson Goodyear's process, was not intended by the patentee and by the Patent Office to be covered by the patent. By Goodyear's patent caoutchouc, or soft rubber, is converted into hard rubber; that is, vulcanized, by mixing sulphur with it in about the proportions of from four ounces to a half pound of the former to a pound of the rubber, and then heating the compound during a period of from three to six hours, to the temperature of from two hundred and sixty to two hundred and seventy-five degrees of Fahrenheit. The use of that process was made indispensable to the invention, and it was the product of that which was the material of which the patented plate is constructed. If, when the patent was granted, there were known substances, other than rubber or caoutchouc, gutta-percha, or gums, that could be vulcanized by the Goodyear process, and converted from a soft into a hard, elastic material, any use of that material for a dental plate might have been an equivalent for the Cummings material, and an infringement of his patent.

This construction of the patent is confirmed by the avowed understanding of the patentee, expressed by him, or on his behalf when his application for the original patent was pending. We do not mean to be understood as asserting that any correspondence between the applicant for a patent and the Commissioner of Patents can be allowed to enlarge, diminish, or vary the language of a patent afterwards issued. Undoubtedly a patent, like any other written instrument, is to be interpreted by its own terms. But when a patent bears on its face a particular construction, inasmuch as the specification and claim are in the words of the patentee, it is reasonable to hold that such a construction may be confirmed by what the patentee said when he was making his application. The understanding of a party to a contract has always been regarded as of some importance in its interpretation.

In his letter to the commissioner, under date of July 30, 1855, written by his attorney, after striking out all reference to gutta-percha, he said: "I beg the office not to consider that because gutta-percha has been used heretofore with artificial teeth, Mr. Cummings' invention is, therefore, the mere substitution of one substance for another and a similar one." The vulcanizing process makes all the difference, and changes the article of manufacture entirely. So in his letter of the same date he amended his specification so as to make it read: "I do not claim the use of gutta-percha, or of any material which is merely rendered plastic by heat and hardened by cooling, in the manufacture of sets of artificial teeth; but what I do claim as my invention, and desire to have secured to me by letters-patent, is the improvement in the manufacture of sets of mineral or other artificial teeth, which consists in combining them with a rubber plate and gums, which (after the insertion of the teeth) are vulcanized by Goodyear's process or any other process." . . .

On a renewed application Dr. Cummings was informed that the office would not object to the admission of a claim limited to the use of vulcanite or hard rubber. In response to this he amended his claim by inserting the word "hard" before "rubber," and also by striking out the word "other" before the words "elastic material," in the claim as previously made, and substituting therefor the words "vulcanite, *i. e.*, an," so as to make it read, "forming the plate and gums in which the teeth are inserted in one piece of hard rubber, or vulcanite, *i. e.*, an elastic material." Thus the patent was granted. In view of this there can be no doubt of what Cummings understood he had patented, and that both he and the commissioner regarded the patent to be for a manufacture made exclusively of vulcanite by the detailed process. We think this, to some extent, at least, tends to confirm the conclusions at which we have arrived in interpreting the patent by its own language. Indeed, we have heretofore expressed doubts whether re-issued letters-patent can be sustained in any case where they contain claims that have once been formally disclaimed by the patentee, or rejected with his acquiescence, and he has consented to such rejection in order to obtain his letters-patent. (Leggett *vs.* Avery, 101 U. S. Reps., 256.) If these doubts be well founded, and the claim of the re-issued letters now before us be, what these complainants insist it is, a claim for the use of any other material than vulcanite, or some substance capable of vulcanization, another question might arise respecting the validity of the re-issue.

We have extended our remarks sufficiently upon this branch of the case. It remains to inquire whether the manufacture, by the defendant, of dental plates, out of the material known as celluloid, or solid collodion, is an infringement of the Cummings re-issue. We think it is not.

Celluloid is a substance of a comparatively recent discovery. Whether it was known at the time Cummings made his invention, or even at the time when his original patent was granted, we do not care now to inquire. It is sufficient for this case that we consider what it is. It is a compound of vegetable fibre, cellulose, or gun-cotton. Undoubtedly it can be employed for manufacturing dental plates, and as a base for artificial teeth. Such a plate may have the fineness, lightness, and elasticity of a plate made of hard rubber by the Cummings process, but it is a substance very different from hard rubber and it is incapable of the same manipulation. It is not vulcanite, and neither it, nor its ingredients are capable of being vulcanized. It contains no sulphur or rubber. None of its constituents are vulcanizing agents. Camphor does not perform the function of sulphur. Under the action of heat its tendency is to soften the compounded mass rather than to harden it, as sulphur does rubber. When the ingredients of celluloid are compounded the product is hard, unlike caoutchouc, or gums generally, and heat softens rather than hardens it. When employed in manufacturing dental plates, the process is wholly unlike that employed in making hard rubber, or vulcanite plates. It is put into a mould, it is true, such as was known and in use before the Cummings invention, but it is put in in a hard state, in its natural condition, aud not soft or plastic, and capable of being pressed around the teeth. The mould cannot be closed until heat is applied. When that is applied the jaws of the mould are gradually screwed together as the celluloid softens, and when the jaws come together the plate is completed. The process requires pressure in addition to heat, in order to reduce the plate to shape, and compress it around the teeth. There is no heating for hours, as is necessary in the vulcanizing process. The work is done in a few minutes. When allowed to cool it is the same hard and bony substance it was before its manipulation, and in this respect also it is unlike vulcanite. It is obvious from all this that neither in the nature of the material of which it is made nor in the process of manufacture, which is an essensial part of the Cummings invention, as we have seen, is the celluloid plate substantially the same as one made of hard rubber.

Nor is celluloid an equivalent for hard rubber, for the reasons already suggested, that it is not capable of vulcanization, and that it cannot be made into a plate by the process prescribed by Cummings. It may be conceded the patentee is protected against equivalents for any part of his invention. He would be, whether he had claimed them or not. But when a product arrived at by certain defined stages or processes is patented, only those things can be considered equivalents for the elements of the manufacture which perform the same function in substantially the same way. The same result may be reached by different processes, each of them patentable, and one process is not infringed by the use of any number of its stages less than all of them.

In view of these considerations we are constrained to rule that a celluloid dental plate is not an infringement of the Cummings patent. Celluloid is not an equivalent for the material which the patent makes essential to the invention, and in the use of it for a dental plate, the process which is inseparable from the invention, is not, and cannot be employed. The decree of the circuit court is, therefore, affirmed.

James H. McKenney,
Clerk Supreme Court U. S.

